# Emerging methods to study bacteriophage infection at the single-cell level

**DOI:** 10.3389/fmicb.2014.00724

**Published:** 2014-12-23

**Authors:** Vinh T. Dang, Matthew B. Sullivan

**Affiliations:** ^1^Department of Ecology and Evolutionary Biology, University of ArizonaTucson, AZ, USA; ^2^Department of Molecular and Cellular Biology, University of ArizonaTucson, AZ, USA

**Keywords:** phage–bacteria interaction, infection strategy, single-cell amplified genome, digital PCR, phageFISH

## Abstract

Bacteria and their viruses (phages) are abundant across diverse ecosystems and their interactions influence global biogeochemical cycles and incidence of disease. Problematically, both classical and metagenomic methods insufficiently assess the host specificity of phages and phage–host infection dynamics in nature. Here we review emerging methods to study phage–host interaction and infection dynamics with a focus on those that offer resolution at the single-cell level. These methods leverage ever-increasing sequence data to identify virus signals from single-cell amplified genome datasets or to produce primers/probes to target particular phage–bacteria pairs (digital PCR and phageFISH), even in complex communities. All three methods enable study of phage infection of uncultured bacteria from environmental samples, while the latter also discriminates between phage–host interaction outcomes (e.g., lytic, chronic, lysogenic) in model systems. Together these techniques enable quantitative, spatiotemporal studies of phage–bacteria interactions from environmental samples of any ecosystem, which will help elucidate and predict the ecological and evolutionary impacts of specific phage–host pairings in nature.

## PHAGE INFECTION OUTCOMES REMAIN LARGELY UNKNOWN FOR UNCULTURED HOSTS

Phages and their bacterial hosts are abundant across diverse ecosystems wherever investigated, including fresh water (Hennes and Simon, 1995), sea water (Bergh, 1989; Fuhrman, 1999; Wommack and Colwell, 2000), sediment (Danovaro et al., 2002), soil (Ashelford et al., 2003; Salifu et al., 2013), and the human mouth, gut, and respiratory tract (Hitch et al., 2004; Letarov and Kulikov, 2009; Willner et al., 2009; Stern et al., 2012; Reyes et al., 2013). Phages influence global biogeochemical cycling by manipulating host populations through mortality, horizontal gene transfer, and viral metabolic reprogramming. First, phage-induced lysis of microbial cells releases organic matter and contributes to carbon, nitrogen, and phosphorus cycling (Shelford et al., 2012; Jover et al., 2014). Second, virus-mediated horizontal gene transfer can have major implications on host evolutionary trajectories. In the oceans, cyanobacterial viruses (cyanophages) have captured core photosystem genes that alter the evolutionary trajectory of these globally distributed photosystems (Lindell et al., 2004; Sullivan et al., 2006). In medicine, prophage-encoded virulence factors routinely transform hosts into pathogens (e.g., *Clostridium botulinum*, *Corynebacterium diphtheria*, *Streptococcus pyogenes*, and *Vibrio cholera*) that cause diseases in humans (Davis et al., 1999; Wagner and Waldor, 2002; Broudy and Fischetti, 2003; Brussow et al., 2004; Mokrousov, 2009). Finally, virus-encoded ‘auxiliary metabolic genes’ (AMGs, *sensu*; Breitbart et al., 2007) can directly alter the metabolic processing of infected cells away from their uninfected states with known implications for photosynthesis (Mann et al., 2003; Dammeyer et al., 2008; Sharon et al., 2009; Thompson et al., 2011, and as above), nearly all of central carbon metabolism (Hurwitz et al., 2013b), and coupled carbon and sulfur cycling (Anantharaman et al., 2014; Roux et al., 2014).

Despite the apparent importance of virus–host interaction outcomes to ecosystem function, our knowledge has been largely bottlenecked by cultivation and technical limitations. Only a fraction (<1%) of microbes in nature grow under typical laboratory conditions (Rappe and Giovannoni, 2003), and few of the 50 known bacterial phyla have cultured phages, largely dominated by three phyla including *Cyanobacteria* (e.g., Suttle and Chan, 1993; Lu et al., 2001; Sullivan et al., 2003), *Proteobacteria* (e.g., Ceyssens et al., 2010; Fogg et al., 2011; Wittmann et al., 2014), and *Bacteroidetes* (e.g., Holmfeldt et al., 2013, 2014). Such model systems are inordinately valuable to test experimental hypotheses and represent the gold standard for developing mechanistic understanding of particular phage–host infection dynamics and outcomes. However, even while new and ecologically abundant phage–host systems are coming into culture (e.g., SAR11 and SAR116 phages; Kang et al., 2013; Zhao et al., 2013), it is unlikely that cultivation-based approaches will be able to map the immense network of phage–host interactions in natural ecosystems.

In addition to establishing reference data representing some fraction of the virosphere, there is a need to better quantify the relative importance of phage–host interaction outcomes in nature. The most commonly described phage life cycles are lytic and lysogenic. Lytic phages infect cells and use host machinery for replicating their nucleic acids (Young, 1992; Catalão et al., 2013). After self-assembly of capsid proteins with their DNA/RNA genomes, host cells are lysed to release 10–100s of progeny into the extracellular environment which then seek to infect other cells. In contrast, temperate phages infect a cell and then either continue with a lytic infection or enter lysogeny whereby the phage chromosome is maintained either integrated in the host’s chromosome or extrachromosomally (Jiang and Paul, 1998; Little, 2005). Under certain conditions (e.g., UV radiation, chemicals, nutrients), temperate phages are induced into the lytic cycle to produce progeny phages and lyse the cells. Lytic phages impact the ecosystem by reducing susceptible host abundances and releasing organic matter from lysed cells. Lysogeny can improve host fitness (Anderson et al., 2011a), including increased growth rate (Edlin et al., 1975), resistance against superinfection by other phages (Bossi et al., 2003), resilience to stressors (Wang et al., 2010), and virulence of a host microbe to its eukaryotic host (Fortier and Sekulovic, 2013).

Other than the lytic and lysogenic cycles, phages are also known to chronically infect their hosts or enter pseudolysogeny. Chronically infecting phages produce progeny that are slowly budded off the cell or passed down to daughter cells without cell lysis at any time (Weinbauer, 2004; Díaz-Muñoz and Koskella, 2014). Similarly, the pseudolysogenic state (sometimes known as “carrier state”), which is poorly understood, implies neither integration of the phage genome into the host genome nor host cell lysis (Weinbauer, 2004; Lood and Collin, 2011; Díaz-Muñoz and Koskella, 2014) and might also be thought of as a chronic infection. This infection strategy is believed to help phages persist in hosts when there is a lack of nutrients to support normal microbial growth. However, both phenomena are insufficiently described in natural communities, and their ecological impacts remain quantitatively unknown at least partly due to the lack of suitable methods.

This leaves three fundamental questions unanswered: (1) *who infects whom*, (2) *how many percent of microbial cells are infected at a particular time point*, and (3) *how does infection progress over time or under different growing conditions*. Fortunately, some of these much-needed methods are beginning to emerge. These include viral tagging (Deng et al., 2012) and viral tagged metagenomics (Deng et al., 2014), large-insert fosmid library screening (Mizuno et al., 2013), and *in silico* linkages derived from sequence composition (Canchaya et al., 2003; Paul, 2008; Akhter et al., 2012) or CRISPR identification (Andersson and Banfield, 2008; Anderson et al., 2011b; Weinberger et al., 2012). In addition, the sequence composition-independent approach of metagenomic analysis (Albertsen et al., 2013) facilitates the recovery of more complete genomes of bacteria, including ones of rare abundance, to allow mining of viral signals at the population level. While all these methods are incredibly powerful for examining population genomic signals across datasets, they lack the ability to develop a single-cell perspective on virus–host interaction outcomes.

Here we review emerging single-cell methods to study phage diversity and infection outcomes with a focus on those that also provide access to uncultured hosts. These methods leverage the sequence information increasingly becoming available to mine virus signals from single-cell genomic datasets and/or to design probes and primers to target particular phage–host groups over time and space in complex communities.

## MINING THE “VIRUS” FROM UNCULTIVATED SINGLE-CELL AMPLIFIED GENOMES (SAGs)

Microbial ecologists are rising to the challenge of understanding the ‘unseen majority’ (Whitman et al., 1998) or ‘microbial dark matter’ (Rinke et al., 2013) by sequencing single-cell amplified genomes (SAGs). This process works by isolating individual cells from an environmental sample (e.g., micro-pipetting, fluorescence-activated cell sorting, microfluidic cell separating), screening those cells using marker gene sequencing, and then amplifying and sequencing the DNA from cells of interest (Lasken, 2012; Macaulay and Voet, 2014). Researchers are generally interested in what metabolisms are associated with this sequence data to pair up known metabolisms with their organismal “owners”. However, such data also represents a treasure trove for new virus discovery. For example, SAG analysis revealed a complete genome of single-stranded DNA virus associated with a cell of one clade, but not with two other marine picobiliphytes (Yoon et al., 2011).

Beyond general surveys, SAGs offer the opportunity for focused ecological and evolutionary study of particular phage–host pairs in nature, which represents a grand challenge in the field and is only achievable using cultures. For example, a recent study (Roux et al., 2014) in a model marine oxygen minimum zone targeted the phages of SUP05 bacteria, an uncultivated group critical in this environment for chemoautotrophy driven by coupled carbon and sulfur nutrient cycling (Wright et al., 2012). A total of 127 SUP05 SAGs were sequenced and mined for viral signal, leading to genomic reference sequences for 69 new phages (Roux et al., 2014). Distributions of detected phages across the SAGs suggested that about one-third of SUP05 cells were infected, with higher infection frequencies where cells are more active. Comparison of these new reference phage genomes to 189 viral or microbial metagenomic datasets suggested that the SUP05 phages were persistent over 3 years in the oxygen minimum zone, but endemic with little indication of the viruses occurring in any of the other datasets available for population-level analysis. Undoubtedly, as more microbial sequence data becomes available, SAGs will provide an invaluable resource for further mapping the virosphere and gaining ecological and evolutionary insight into specific phage–host interaction dynamics.

## LINKING VIRUSES TO THEIR HOSTS USING DIGITAL PCR

Digital PCR was initially used to quantify the fraction of DNA molecules with mutations predefined in cancer cells (Vogelstein and Kinzler, 1999). Briefly, genomic DNA is diluted to extinction in microtiter plates (e.g., 96- or 384-well plates) so that individual templates can be separately PCR-amplified. This enables a rare mutant template to be detected from the mixture with sensitivity and accuracy higher than the ∼2-fold detection limits possible with quantitative real-time PCR (Smith and Osborn, 2009; Baker, 2012). Based on a fluorescence measurement, the mutant signal can be distinguished from the wild-type by a loop sequence of fluorescent probe molecular beacon-RED (MB-RED, 5′-Cy3-oligonucleotide probe-Dabcyl-3′) that detects wild-type and mutant products as compared to an MB-GREEN (5′-fluorescein-oligonucleotide probe-Dabcyl-3′) probe that only recognizes wild-type template as mutations impede probe hybridization.

**FIGURE 1:**
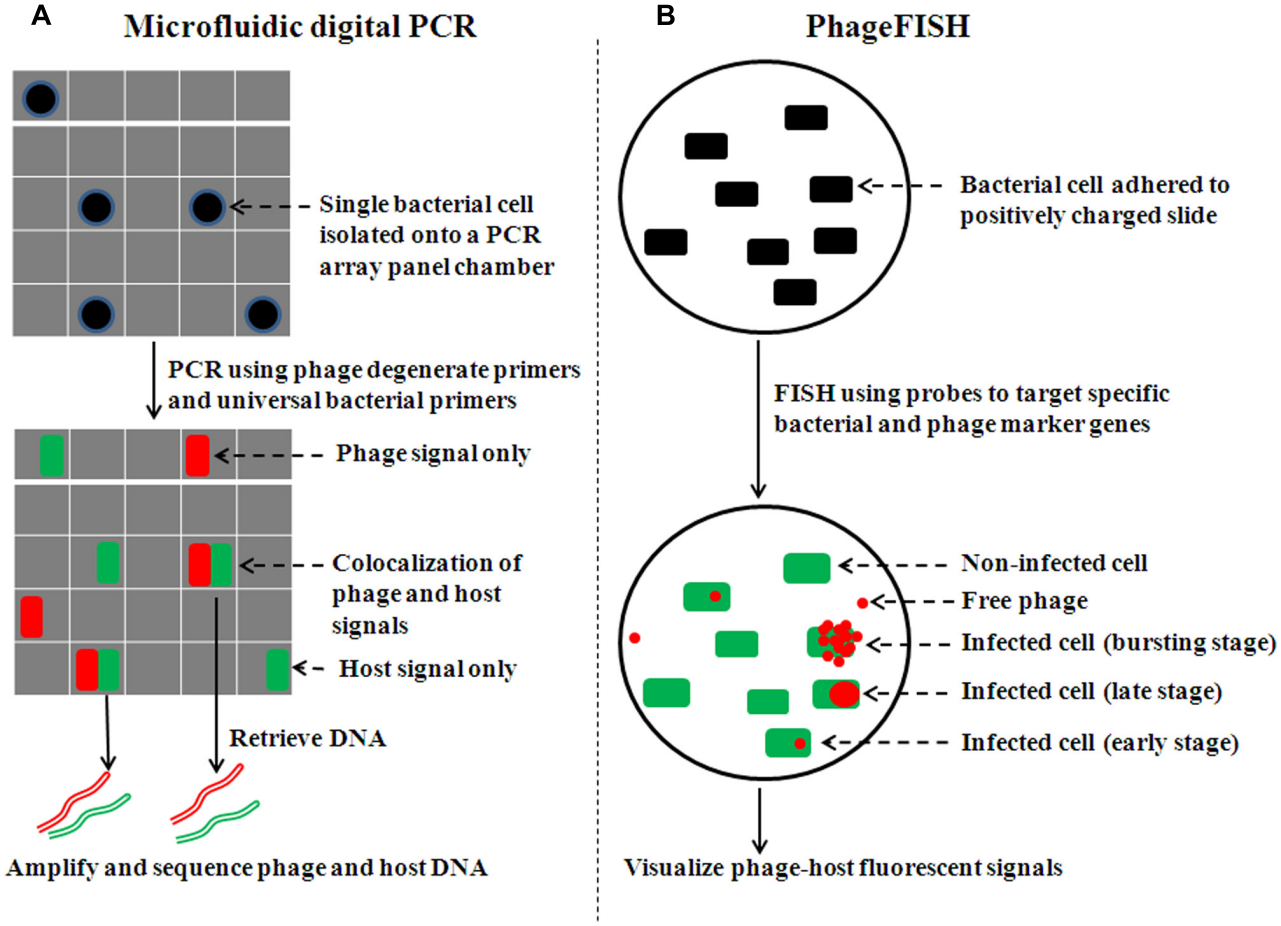
**Overview of procedures of single-cell experimental methods to examine phage–host interactions. (A)** For microfluidic digital PCR, cells are sorted onto an array panel with the majority of chambers containing no or single cells (adapted from Tadmor et al., 2011). Concurrent amplification is carried out for both phage and bacterial marker genes. Co-localization of phage and bacterial signals is shown in FAM and HEX channels, respectively, with fluorescence in half of each PCR array chamber. **(B)** For phageFISH, the phage–host sample is either immobilized on 0.2 μm filter membranes or adhered to positively charged slides (adapted from Allers et al., 2013). A bacterial marker gene (16S rRNA) is detected by oligoprobes conjugated to horseradish peroxidase (HRP) molecules, which catalyze the deposition of many fluorescently labeled tyramides (e.g., green Alexa_488_). Subsequently, the phage marker gene is detected by a set of double-stranded DNA probes (6–12) labeled with digoxigenin (DIG) molecules. DIG is then recognized by an antibody that is labeled with HRP molecule to catalyze the deposition of many fluorescently labeled tyramides (e.g., red Alexa_595_).

Application of microfluidic technology improves digital PCR (Ottesen et al., 2006; Marcy et al., 2007; Zare and Kim, 2010) by enabling larger-scale study (e.g., isolate and analyze single cells on a 765-chamber PCR array panel, where most chambers contain no or one cell; **Figure 1A**). When complemented with sequencing, microfluidic digital PCR has helped elucidate phage–host associations from environmental samples (e.g., termite hindgut; Tadmor et al., 2011), specifically answering two critical research questions in viral and microbial ecology: who infects whom, and what percent of particular host cells is infected by a particular phage. Pragmatically, the phage–host association is revealed by co-localized fluorescent signals (FAM and HEX for phage and host, respectively). PCR products of a phage conserved gene (e.g., terminase) hybridize to FAM-labeled probe while that of bacterial 16S rRNA gene bind to HEX-labeled probe. Finally, phage and host can be identified by sequencing DNA retrieved from PCR array chambers with co-localized signals.

The critical step in applying microfluidic digital PCR to other systems is the design of appropriate primers. In the termite hindgut work (Tadmor et al., 2011), universal 16S rRNA primers and terminase primers were used to identify the host and phage, respectively. New primer sets can be designed by analyzing metagenomic datasets (e.g., use of Metagenome Cluster Analysis Tool; Tadmor et al., 2011) to identify lineage-specific marker genes for particular viral and microbial targets. The program CODEHOP (Rose et al., 2003) can then explore marker gene regions to design primers with minimal degeneracy and primer dimers, as well as melting temperature profiles similar to those of the bacterial rRNA universal primer set to enable co-amplification. Several sets of designed primers are then experimentally tested to optimize amplification performance and detection limit (e.g., <100 gene copies; Tadmor et al., 2011).

There are a few challenges for linking viruses to their hosts by microfluidic digital PCR. First, false positive signals can occur from multiple phage genes released from prematurely lysed cells, multiple bacterial 16S rRNA genes from cells adhered to the same chamber, or fluorescence signal spilled over from neighboring chambers (Tadmor et al., 2011). These issues can be circumvented by excluding chambers with multiple bacterial and/or viral signals, which notably rules out co-infections whose frequencies are largely unknown, and considering only chambers flanked by ones with no fluorescence in both channels. Alternatively, a barcoding strategy can be applied so that individual DNA template can be tagged with a unique barcode, amplified, and read by sequencing (Kinde et al., 2011). Second, the 765-chamber PCR array panel, while already a large increase in throughput, is still likely only a tiny sub-sample of naturally occurring diversity. One way forward would be adoption of droplet-based strategies (Hindson et al., 2011; Jones et al., 2014) to sort cells into nanoliter-sized droplets (QX100/QX200 Droplet Digital PCR System, Bio-Rad) where PCR could occur on scales of 10s of 1000s of reactions while leveraging automated fluidic systems. Such data scales would allow researchers to examine specific phage–host pairs with a very small fraction of cells and/or phages in environmental samples.

## LINKING AND VISUALIZING PHAGE–HOST INTERACTIONS USING FLUORESCENCE *IN SITU* HYBRIDIZATION

Alternative to digital PCR-based methods, fluorescence *in situ* hybridization (FISH) methods offer an opportunity to examine specific phage–host interactions. GeneFISH (Moraru et al., 2010) was originally developed to detect cellular marker genes at the single-cell level. Briefly, microbial communities are collected (e.g., on 0.2 μm membrane filters or positive-charged slides), and then probes are introduced to target a bacterial marker gene (e.g., 16s rRNA) and a biogeochemically relevant gene. Horseradish peroxidase (HRP) molecules linked to probes catalyze the deposition of many fluorescently labeled tyramides so that both gene targets are represented by different fluorescent signals (e.g., green Alexa_488_ and red Alexa_595_), again enabling microscopy-based co-localization of the separate gene targets. Here the goal was to find the microbial “owners” of biogeochemically important genes from samples of complex communities.

**FIGURE 2:**
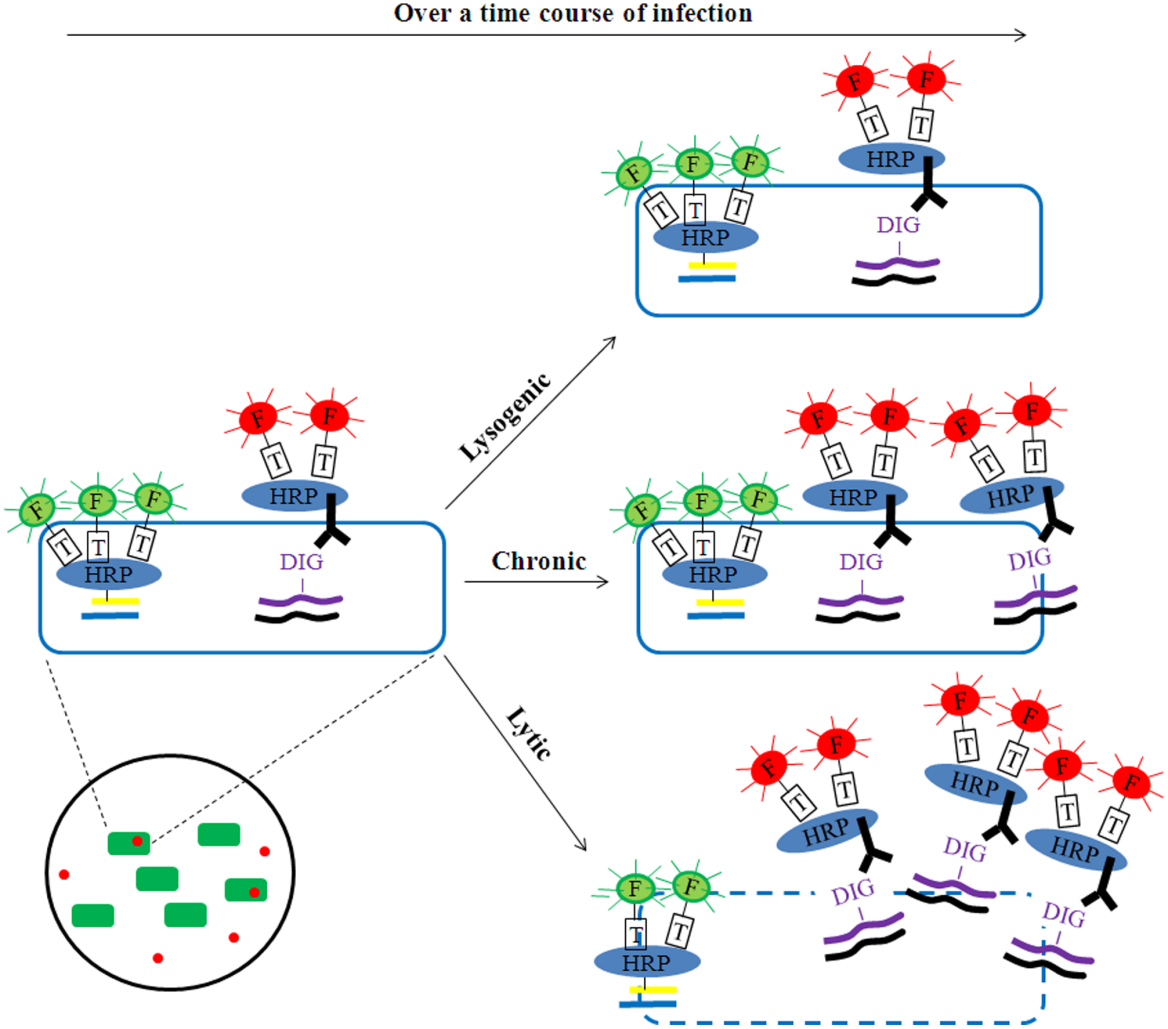
**Potential application of phageFISH to identify modes of infection.** Over a time course of infection, phages could be detected as lytic (e.g., scattering-out of phage signal together with reduction/loss of bacterial signal), chronic (e.g., gradual increase of phage signal together with no reduction/loss of bacterial signal), or lysogenic (e.g., no change of phage signal). The phage signal intensity (red) would remain constant for lysogenic infections, while it would increase substantially for lytic infections and to a lesser extent for chronic infections. The bacterial signal (green) would decrease over time for lytic infection as a result of cell lysis and the release of phage progeny, while it should remain unchanged in chronic infections due to the production of progeny shredded out slowly without damaging cell membrane integrity. In this way the dual-labeling signals can help discriminate three modes of phage infection at the single-cell level.

Building upon these findings, phageFISH (Allers et al., 2013) was developed to more sensitively target marker genes of both cells and their infecting phages. It improved upon previous geneFISH and FISH-based methods (Hoshino and Schramm, 2010; Kawakami et al., 2010; Moraru et al., 2010) by increasing detection efficiency of phage genes from less than 40% to 98% by using more probes (up to 12 probes, ∼300bp each) to enable detection of a single phage gene copy within a cell. Though long probes (∼800 bp) have been used to achieve similar detection efficiency (Kawakami et al., 2012), the binding specificity for naturally occuring phage targets can be affected due to high genetic variation where such long stretches of conservation are uncommon. This high sensitivity enables phageFISH to measure infection dynamics (**Figure 1B**) from early (single phage template) to late/bursting stage (multiple phage templates encapsulated and spread out), as demonstrated in marine podovirus–gammaproteobacterial host model system (Allers et al., 2013). Such measurements are invaluable for discerning among lytic, lysogenic, and chronic phage infection modes (**Figure 2**), and phageFISH is the only method available to do this without genetics.

For single phage–host model systems, the production of probe sets is straightforward with identification of 6–12 gene regions with similar %G+C and length to achieve melting temperatures that range no more than 1–2°C. However, for environmental samples, bioinformatics is required to identify suitable areas of conservation as even core orthologous genes in viruses can be <40% identical for 21 out of 57 tested viral taxa (Kristensen et al., 2013). Growing viral metagenomic datasets [e.g., viromes from seawater (Hurwitz and Sullivan, 2013), freshwater (Roux et al., 2012), marine sediment (Breitbart et al., 2004; Yoshida et al., 2013), and human gut (Waller et al., 2014)] should at least provide the sequence data to identify highly conserved phage gene targets suitable for phageFISH probe design. Also well-studied phage groups like T4-like myoviruses (Sullivan et al., 2010; Deng et al., 2014) and T7-like podoviruses (Labrie et al., 2013), for which “core genomes” are already identified, offer prime starting materials for phageFISH probe development and application. Pragmatically, probes must target all phages of interest with no more than 5% mismatches to be effective (Moraru et al., 2010), which requires consideration of appropriate sub-groups to target.

In addition to probe design challenges, the use of a catalyzed reporter deposition (CARD) step limits phageFISH to at best “relative quantification.” Specifically, phageFISH cannot absolutely quantify phage targets within cells where tyrosine molecules of close proximity to target-bound probes become limited. To acquire absolute quantification of per-cell phage signal, the CARD step needs to be eliminated, perhaps replaced by super-resolution microscopy to allow sensitive signal detection (Huang et al., 2010; Schermelleh et al., 2010; Vaughan et al., 2012). Finally, phageFISH is currently, relatively low throughput since only a limited number of samples on membrane filters or positive slides can be handled at a time. To increase throughput, especially for experiments comparing infection of one phage on multiple hosts or different phages on the same host, phageFISH samples could be prepared in a 96- or 384-well plate format and analyzed by an automated imaging system.

## CONCLUSION

These three emerging methods enable future studies to examine phage–host interaction at the single-cell level with particular strengths in accessing the uncultivated phage–host pairs in nature. Mining the viral signal from rapidly growing SAG datasets offers a high-throughput informatic approach to identify phage–host pairs and temperate phages, as well as estimate the frequency of infection in microbial populations. Complementarily, digital PCR and phageFISH utilize single-cell resolution marker gene tracking of specific phage–host pairs through space and time with strong potential for high-throughput adaptation that would allow more rapid screening and large-scale experimental tracking. While microfluidic digital PCR likely has more immediate high-throughput capability, phageFISH offers the sole ability to discriminate between infection strategies (e.g., lytic, chronic, lysogenic) through single-cell dynamic measurements. To further understand particular viral gene dynamics or the function of any viral-encoded genes, gene or protein expression studies are required during the course of infection (e.g., Lindell et al., 2007; Dammeyer et al., 2008; Thompson et al., 2011). Together these and other advances in viromics (e.g., Andrews-Pfannkoch et al., 2010; John et al., 2011; Duhaime et al., 2012; Hurwitz et al., 2013a; Culley et al., 2014), informatics (e.g., Jiang et al., 2012; Albertsen et al., 2013; Gagic et al., 2014; Hurwitz et al., 2014), and theory (e.g., Beckett and Williams, 2013; Weitz et al., 2013; Soffer et al., 2014; Thingstad et al., 2014) are transforming our ability to explore natural viral communities at the single-cell and whole population levels and increasingly in their hosts. While this review is focused on phages, relevant archaeal hosts and their viruses could also be investigated using these methods. These advances should help viral and microbial ecologists begin to develop predictive models for these critical ecological and evolutionary cogs in natural ecosystems.

## Conflict of Interest Statement

The authors declare that the research was conducted in the absence of any commercial or financial relationships that could be construed as a potential conflict of interest.
